# Effectiveness of Contemporary Invisalign® Clear Aligners for Derotation and Deep Bite Correction of Different Degrees of Severity in Adolescents and Adults: A Retrospective Clinical Study

**DOI:** 10.7759/cureus.116953

**Published:** 2026-09-26

**Authors:** Shweta Chhaya, Tanvi Sharma, Supriya Palande, Swati Pakolu, Pranali Agawane, Meghna Vandekar

**Affiliations:** 1 Department of Orthodontics and Dentofacial Orthopedics, Dr. G.D. Pol Dental College and Hospital, PG Institute, Navi Mumbai, IND

**Keywords:** clear aligners, deep bite, orthodontic tooth movement, tooth rotation, treatment outcome

## Abstract

Introduction

Clear aligners have evolved substantially with improvements in aligner materials, digital treatment planning, and optimized attachments. However, the predictability of complex movements such as tooth derotation and deep bite correction remains variable. This study evaluated the effectiveness of contemporary Invisalign® clear aligners (Align Technology, Inc., Tempe, AZ) for these movements and examined factors associated with treatment accuracy.

Materials and methods

This retrospective clinical study included 50 patients treated with contemporary Invisalign® clear aligners. Twenty-five patients were classified as growing and 25 as non-growing. Treatment accuracy was determined by comparing the predicted and achieved tooth movements using a digital model assessment. Derotation accuracy was evaluated according to the growth status, rotational severity, tooth type, and dental arch. Deep bite correction accuracy was assessed according to growth status, severity, anterior and posterior attachments, incisor proclination, and posterior extrusion. Non-parametric statistical tests were used, with statistical significance set at p < 0.05.

Results

Derotation accuracy did not differ significantly between growing and non-growing patients (82.61% vs. 83.59%; p = 0.218). Accuracy differed significantly according to rotational severity (p < 0.001), with median values of 88.83%, 78.61%, and 61.77% for mild, moderate, and severe rotation, respectively. The difference in tooth type was also significant (p = 0.005). Deep bite correction accuracy did not differ according to growth status (69.0% vs. 61.5%; p = 0.633) but differed significantly according to severity (p = 0.026), with median accuracies of 100.0%, 66.7%, and 57.5% for mild, moderate, and severe cases, respectively. For deep bite correction, the severity of the initial deep bite and mandibular incisor proclination was significantly associated with treatment accuracy.

Conclusion

Contemporary Invisalign® clear aligners were effective for derotation and deep bite correction, although their accuracy decreased with increasing treatment severity.

## Introduction

Clear aligner therapy has evolved considerably with advances in digital orthodontic technology, three-dimensional imaging, computer-aided treatment planning, and additive manufacturing [1]. The increasing demand for esthetic orthodontic appliances, particularly among adults, has contributed to the widespread adoption of clear aligners as alternatives to conventional fixed appliances. Their advantages include improved aesthetics, comfort, ease of use, and potentially better oral hygiene, making them an attractive treatment modality for both adolescent and adult patients [2]. The development of digital treatment planning has further enabled clinicians to visualize sequential tooth movements and compare planned tooth positions with treatment outcomes [3].

Clear aligner systems have progressed through different generations, with third-generation systems incorporating optimized attachments designed to improve the biomechanical control of specific tooth movements. Invisalign® (Align Technology, Inc., Tempe, AZ) is a third-generation clear aligner system that incorporates SmartForce® optimized attachments, SmartStage® technology, and SmartTrack® material [4]. These developments were intended to enhance the predictability of complex movements such as tooth rotation and deep bite correction [3]. Contemporary clear aligner therapy has demonstrated clinically meaningful but modest effectiveness in deep bite correction, with treatment effects occurring predominantly through dentoalveolar mechanisms such as mandibular incisor intrusion and limited molar extrusion. However, considerable variability in treatment response remains, particularly with more severe deep bites, highlighting the need for careful case selection and biomechanical planning [5,6].

Although clear aligner therapy has become increasingly popular for rotational tooth movements, its predictability remains variable and is influenced by tooth morphology and treatment mechanics [7]. A recent systematic review reported a wide range of rotational accuracies across studies, with incisors generally demonstrating greater predictability than maxillary canines and premolars, highlighting the continuing challenges associated with complex rotational movements [8]. The derotation accuracy has been reported to decrease with increasing rotational requirements, whereas attachments can improve rotational control [8]. Deep bite correction may involve a combination of true anterior intrusion, pseudo-intrusion, and posterior extrusion, making the assessment of the achieved movement particularly relevant.

Therefore, the present study aimed primarily to evaluate the percentage accuracy of derotation and deep-bite correction by comparing ClinCheck® (Align Technology, Inc., Tempe, AZ)-predicted tooth positions with achieved treatment outcomes. Secondary objectives were to assess treatment accuracy according to growth status and movement severity, and to evaluate subgroup differences according to tooth type and dental arch for derotation and according to anterior and posterior attachment status, maxillary and mandibular proclination, and posterior extrusion for deep-bite correction. The association between patient age and the degree of achieved tooth movement was also assessed.

## Materials and methods

Study design and setting

This retrospective clinical study was conducted in the Department of Orthodontics and Dentofacial Orthopedics, Dr. G.D. Pol Dental College and Hospital, PG Institute (previously known as Y.M.T. Dental College and Hospital), Navi Mumbai, India. This study was based on the retrospectively available orthodontic treatment records of patients who had undergone treatment exclusively with third-generation clear aligners. The study records were obtained from the Department of Orthodontics, where treatment was provided under the supervision of the same orthodontist who was an Invisalign® provider. The orthodontic treatment records included in this study covered the period from January 2018 to December 2020. The analysis was restricted to the original series of aligners without the incorporation of subsequent refinement treatment into the primary treatment sequence. Ethical clearance for the study was obtained from the ethical committee (YMTDC&H/IEC/2021/021), and the required permissions were obtained from the concerned authorities before commencement of the study. As this study involved a retrospective analysis of existing treatment records, patient-related information was handled confidentially. Invisalign® providers also provided the required consent for the retrieval of treatment records from the Invisalign® doctor platform.

Study population

The study population comprised patients aged 10-45 years who had undergone contemporary Invisalign® clear aligners treatment and had at least one of the two treatment objectives under investigation: derotation of a tooth exceeding 5° or correction of an initial deep bite exceeding 2 mm. The study included 50 patients, with 25 classified as growing and 25 as non-growing. Growth status was determined from pre-treatment lateral cephalometric radiographs using the cervical vertebral maturation index (CVMI) method [9]. The morphology and maturation stages of the cervical vertebrae were assessed using a lateral cephalogram and classified according to the CVMI staging system. Patients with CVMI stages I-IV were classified as growing, whereas those with CVMI stages V or VI were classified as non-growing. Growth status was subsequently used as an analytical variable to determine whether the treatment accuracy differed between growing and non-growing patients.

Eligibility criteria

Patients aged 10-45 years were eligible for inclusion if they required at least one of the predefined treatment objectives, namely, derotation of one or more teeth by more than 5° or correction of an initial deep bite greater than 2 mm. The patients were required to be in good general and dental health and have no contraindications to orthodontic treatment. The manufacturer's recommended protocol for changing the aligners at weekly intervals must be followed. Patients who underwent combined treatment with fixed edgewise appliances were excluded. Cases in which special instructions were incorporated into ClinCheck® to modify the treatment rate were excluded.

Mid-course corrections or refinements were excluded from the primary treatment assessment because their incorporation would have changed the baseline tooth position and potentially reduced the magnitude of the tooth movement being evaluated. Minimal molar movement in all three spatial planes was required because the molars served as relatively stable reference structures during the digital superimposition. Patients were excluded if they were considered non-compliant, had cleft lip and/or palate or other craniofacial anomalies, or had medical conditions that compromised their suitability for orthodontic treatment.

Contemporary Invisalign® clear aligners treatment

All the patients were treated using Invisalign®. The study included patients treated with the contemporary Invisalign® clear aligners system incorporating SmartTrack® aligner material and the G5 deep bite protocol [4]. Treatment planning was performed by treating orthodontists using the ClinCheck® software. The treatment plan was developed according to the clinician's professional judgment without imposing a study-specific restriction on the type or placement of attachments. Available attachment configurations include optimized attachments, conventional attachments, and cases without attachments. The aligners were changed weekly, according to the treatment protocol. The mean duration of treatment was 36 weeks.

Digital treatment records

Retrospective records were obtained from the Invisalign® Doctor Platform with appropriate authorization from the Invisalign® providers. Digital treatment models were exported from ClinCheck® in stereolithography (STL) format. For each case, the initial STL model from the primary ClinCheck® treatment plan was designated the initial model, whereas the final STL model from the primary ClinCheck® sequence was designated the predicted model. The initial STL model obtained from ClinCheck® refinement was designated as the achieved model because it represented the actual tooth position following completion of the original aligner sequence. Refinement treatment itself was not incorporated as an intervention in the analysis.

Digital model superimposition

The initial, predicted, and achieved three-dimensional digital models were superimposed using the 3D Slicer software, version 4.10.2 (3D Slicer; Kitware, Inc., Clifton Park, NY). The built-in best-fit superimposition algorithm was used to register the digital models. Stable anatomical landmarks were identified on the superimposed models, and rotational measurements were obtained relative to a true vertical reference drawn in the sagittal plane through the midpalatal raphe. The initial and predicted models were superimposed to determine the baseline severity of rotation, and the predicted and achieved models were compared to determine the predicted and achieved rotational corrections (ARCs) (Figure 1).

**Figure 1:**
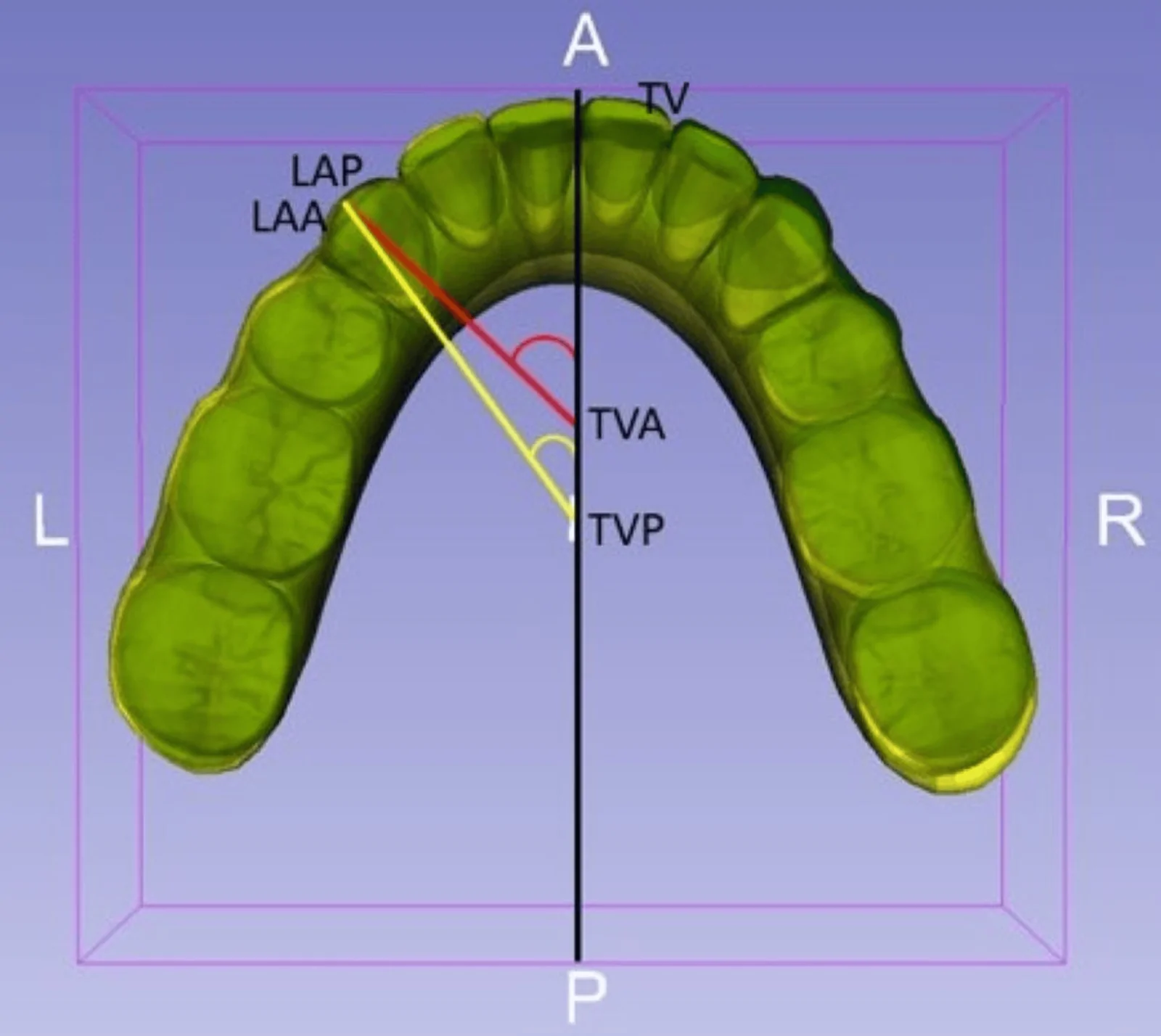
Assessment of rotation in left mandibular canine tooth with achieved (red line angle) against predicted (yellow line angle). LAA: long axis of the achieved tooth position, LAP: long axis of the predicted tooth position, TV: true vertical, TVA: point of intersection of long axis and true vertical for achieved outcome, TVP: point of intersection of long axis and true vertical for predicted outcome Three-dimensional digital models were superimposed using 3D Slicer software, version 4.10.2, to assess tooth rotation relative to the transverse and anteroposterior reference axes.

Assessment of rotational severity and accuracy

Initial rotational discrepancy was assessed using three-dimensional digital models and categorized as mild (5-20°), moderate (21-35°), or severe (>35°). Rotational displacement was measured as the angle between the long axis of the tooth and the true vertical reference on the corresponding digital model. The initial, predicted, and achieved rotational positions are recorded for each case. The ARC was calculated as the difference between the pre-treatment and post-treatment rotational measurements: 

\begin{equation} \text{ARC} = \text{Pre-treatment rotation} - \text{Post-treatment rotation} \label{eq:arc_text} \end{equation}

Predicted rotational correction (PRC) was defined as the amount of rotation programmed in the ClinCheck® treatment plan. The percentage accuracy of rotational correction was calculated as follows:

\begin{equation} \text{Accuracy (%)} = 100 \times \left( 1 - \frac{PRC - ARC}{PRC} \right) \end{equation}

The variables recorded included tooth type, age, growth status, pre-treatment rotation, predicted and achieved post-treatment rotation, predicted and achieved rotational correction, percentage accuracy of the predicted treatment outcome, rotational severity, attachment status, and number of aligners. When more than one tooth in the same patient required rotational correction, the tooth with the greatest baseline rotational discrepancy was designated as the index tooth. Only the index tooth was included in the primary analysis to avoid correlated observations within patients and to maintain statistical independence.

Assessment of deep bite severity and correction accuracy

The initial deep bite severity was classified a priori according to the pre-treatment overbite measurement as mild (≤2.5 mm), moderate (2.6-4.0 mm), or severe (>4.1 mm). Pre-treatment overbite, predicted overbite correction, and post-treatment overbite were measured using the Tooth Measure function of ClinCheck® software. Achieved bite correction (ABC) was calculated as the difference between pre-treatment and post-treatment overbite as follows, whereas predicted bite correction (PBC) represented the amount of correction programmed in the ClinCheck® treatment plan. The percentage accuracy of deep bite correction was calculated as follows:

\begin{equation} \text{Accuracy (%)} = 100 \times \left( 1 - \frac{PBC - ABC}{PBC} \right) \end{equation}

The analysis included initial deep bite severity and growth status, along with treatment-related variables, including anterior and posterior attachments, maxillary and mandibular proclination, and posterior extrusion.

Assessment of true intrusion and incisor proclination

Pre-treatment and post-treatment lateral cephalograms available in the treatment records were used to evaluate the vertical and angular changes associated with deep bite correction. Maxillary and mandibular superimpositions were performed using the respective reference structures. The maxilla was superimposed on the palatal plane, whereas the mandible was superimposed on the mandibular plane with reference to the inner surface of the symphysis. True incisor intrusion was defined as the linear displacement of the center of resistance of the maxillary or mandibular incisors between the initial and final positions. Lower incisor inclination, representing pseudo-intrusion through incisor proclination, was assessed using the incisor mandibular plane angle (IMPA), which is defined as the angle formed by the long axis of the mandibular central incisor and the mandibular plane formed by the lower border of the mandible.

Assessment of posterior extrusion

Posterior extrusion was evaluated using initial, predicted, and digital models. Premolar extrusion was defined as bodily movement of the premolar toward the occlusal plane and was measured at the buccal cusp tip. Corresponding measurements were obtained using digital model superimpositions.

Attachment assessment

The attachment status was recorded using the ClinCheck® treatment plan. Cases were categorized according to the presence of optimized attachments, conventional attachments, or the absence of attachments. For deep bite correction, the presence or absence of the anterior and posterior attachments was also recorded. The attachment status was subsequently examined as a potential factor influencing the accuracy of derotation and deep bite correction.

Data recording and variables

The following variables were extracted from the retrospective records: age, sex, growth status, treatment duration, number of aligners used, initial and post-treatment rotation, initial and post-treatment overbite, severity of rotation, severity of deep bite, tooth type, dental arch, attachment status, anterior and posterior attachment status, maxillary and mandibular proclination, true incisor intrusion, and posterior extrusion. The primary outcome variables were the percentage accuracy of derotation and percentage accuracy of deep bite correction. All the measurements were entered into a structured datasheet. To minimize potential errors during data extraction, the definitions of the predicted and achieved movements were kept consistent across cases, with the predicted position derived from the original ClinCheck® plan and the achieved position derived from the post-treatment/refinement scan representing the outcome of the original aligner series.

Sample size calculation

An a priori sample-size calculation was performed using G*Power software, version 3.1.9.7 (Heinrich-Heine-Universität Düsseldorf, Düsseldorf, Germany). The calculation was based on an independent-samples t-test, assuming a medium-to-large effect size of 0.70, 80% statistical power, and a two-sided alpha level of 0.05. The calculation yielded the minimum required sample size of 44 patients, corresponding to 22 patients per group. Allowing for approximately 10% loss of data or incomplete records, the final required sample size was increased to 50 patients, with 25 patients allocated to each analytical growth status group.

Statistical analysis

The collected data were subsequently analyzed using IBM SPSS Statistics (version 25.0 (IBM Corp., Armonk, NY)). The Shapiro-Wilk test was used to assess the normality of continuous variables. As the continuous outcome variables did not follow a normal distribution, non-parametric statistical methods were used for primary analyses. Continuous variables are summarized as median and interquartile range (IQR), while categorical variables are presented as frequencies and percentages. The Mann-Whitney U test was used for comparisons between two independent groups. Comparisons involving more than two independent groups were performed using the Kruskal-Wallis test. When a statistically significant Kruskal-Wallis result was obtained, Dunn's post-hoc test with Bonferroni adjustment was performed to identify the specific group differences responsible for the overall significant result. The effect sizes have been reported as applicable. A two-tailed p-value < 0.05 was considered statistically significant. For rotational analysis, the percentage accuracy was compared according to the growth status, severity of rotation, tooth type, and dental arch. For deep bite analysis, the percentage accuracy was compared according to growth status, severity of overbite, anterior and posterior attachment status, maxillary and mandibular proclination, and posterior extrusion. The use of a single index tooth per patient for the primary rotational analysis was intended to prevent intrapatient clustering and maintain the assumption of statistical independence.

## Results

The final analytical sample comprised 50 patients, including 25 (50%) in the growing group and 25 (50%) in the non-growing group. Of the total sample, 28 (56%) were males, and 22 (44%) were females. The mean age of the study population was 20.50 ± 9.39 years, with mean ages of 12.50 ± 4.21 years in the growing group and 28.50 ± 5.20 years in the non-growing group. A statistically significant difference in age was observed between the two groups (p < 0.001). Baseline rotation was also significantly different between the growing and non-growing groups (p = 0.015), whereas post-treatment rotation was not (p = 0.629). No significant differences were observed in sex, pre-treatment deep bite, post-treatment deep bite, or treatment duration. However, the number of aligners used was significantly greater in the growing group than in the non-growing group (p = 0.007). Demographic and baseline characteristics of the study population are shown in Table 1.

**Table 1:** Demographic and baseline characteristics of the study population. Data are presented as mean ± standard deviation (SD) for normally distributed continuous variables, median (interquartile range (IQR)) for non-normally distributed continuous variables, and number (percentage) for categorical variables. An independent-samples t-test was used for normally distributed continuous variables, and the Mann-Whitney U test was used for non-normally distributed continuous variables; the χ² test was used for categorical variables. *p < 0.05 was considered statistically significant.

Parameter	Category	Overall (n = 50)	Growing (n = 25)	Non-growing (n = 25)	Test statistic	p-value
Age, mean ± SD	Years	20.50 ± 9.39	12.50 ± 4.21	28.50 ± 5.20	t = 11.96	< 0.001*
Sex, n (%)	Males	28 (56%)	13 (52%)	15 (60%)	χ² = 0.32	0.569
	Females	22 (44%)	12 (48%)	10 (40%)		
Rotation, median (IQR), ° (degree)	Pre-treatment	60.0 (52.0-71.5)	68.0 (55.0-74.0)	52.0 (45.0-60.0)	U = 180.5	0.015*
	Post-treatment	10.0 (7.2-17.0)	11.0 (8.0-17.0)	10.0 (7.0-17.0)	U = 279.5	0.629
Deep bite, median (IQR), mm	Pre-treatment	4.3 (4.0-6.2)	4.3 (4.0-6.8)	4.3 (4.0-5.5)	U = 270.5	0.421
	Post-treatment	2.0 (1.2-2.0)	2.0 (1.5-3.0)	1.8 (1.0-2.0)	U = 229.5	0.101
Treatment duration, mean ± SD	Months	10.80 ± 4.26	10.56 ± 4.38	11.04 ± 4.14	t = -0.40	0.692
Aligners used, mean ± SD	Units	30.34 ± 10.30	34.20 ± 9.79	26.48 ± 9.47	t = 2.83	0.007*

Accuracy of derotation

The primary analysis of derotation accuracy demonstrated no statistically significant difference between the growing and non-growing patients. The median derotation accuracy was 82.61% (IQR, 77.74-90.70) in the growing group and 83.59% (IQR, 76.92-89.73) in the non-growing group (p = 0.218; r = 0.07). The severity of rotation showed a highly significant association with derotation accuracy (p < 0.001; η² = 0.77). The median accuracy decreased from 88.83% (IQR, 83.76-92.25) in mild cases to 78.61% (IQR, 76.06-80.49) in moderate cases and 61.77% (IQR, 52.33-63.62) in severe cases. Tooth type was also significantly associated with derotation accuracy (p = 0.005; η² = 0.25), with median accuracies of 86.97% (IQR, 80.59-92.63) for canines, 76.92% (IQR, 62.68-83.22) for incisors, and 83.59% (IQR, 80.80-90.70) for premolars. No significant difference was observed between the maxillary and mandibular arches (p = 0.724; r = 0.05). The results are presented in Table 2.

**Table 2:** Primary analysis of derotation accuracy according to patient and treatment characteristics. Data are presented as number (percentage) for categorical variables and median (interquartile range (IQR)) for derotation accuracy. The Mann-Whitney U test was used for comparisons between two independent groups, and the Kruskal-Wallis test was used for comparisons among more than two independent groups. Significant Kruskal-Wallis results were followed by Dunn’s post hoc test with Bonferroni adjustment; *p < 0.05 was considered statistically significant. η²: eta-squared effect size, r: rank-biserial effect size.

Factor	Category	n (%)	Median (IQR), %	Test statistic	p-value	Effect size
Growth pattern	Growing	25 (50%)	82.61 (77.74-90.70)	U = 241.5	0.218	r = 0.07
	Non-growing	25 (50%)	83.59 (76.92-89.73)			
Severity of rotation	Mild	29 (58%)	88.83 (83.76-92.25)	H = 36.41	< 0.001*	η² = 0.77
	Moderate	14 (28%)	78.61 (76.06-80.49)			
	Severe	7 (14%)	61.77 (52.33-63.62)			
Tooth type	Canine	13 (26%)	86.97 (80.59-92.63)	H = 10.27	0.005*	η² = 0.25
	Incisor	15 (30%)	76.92 (62.68-83.22)			
	Premolar	22 (44%)	83.59 (80.80-90.70)			
Arch	Maxillary	28 (56%)	83.85 (77.51-90.70)	U = 289.5	0.724	r = 0.05
	Mandibular	22 (44%)	81.29 (77.56-89.51)			

Post-hoc pairwise comparisons using Bonferroni adjustment demonstrated a statistically significant difference in the accuracy of derotation between the incisors and premolars (adjusted p = 0.005). Comparisons between the incisors and canines (adjusted p = 0.118) and between the premolars and canines (adjusted p = 1.000) were not statistically significant. For rotational severity, significant differences were observed between moderate and mild cases (adjusted p < 0.001) and between severe and mild cases (adjusted p < 0.001), whereas the difference between moderate and severe cases was not significant (adjusted p = 0.292). The post-hoc comparisons are presented in Table 3.

**Table 3:** Bonferroni-adjusted post-hoc pairwise comparisons of derotation accuracy. Pairwise comparisons were performed using Dunn’s post-hoc test following a significant Kruskal-Wallis test, with Bonferroni adjustment for multiple comparisons. *p < 0.05 was considered statistically significant. Adjusted p-values are Bonferroni-corrected.

Factor	Pairwise comparison	Mean difference	Test statistic	p-value	Adjusted p-value
Tooth type	Incisor vs. premolar	-14.79	-15.47	< 0.001*	0.005*
	Incisor vs. canine	-12.37	-11.38	0.014*	0.118
	Premolar vs. canine	2.42	4.09	0.406	1.000
Severity of rotation	Moderate vs. severe	23.18	11.18	0.097	0.292
	Moderate vs. mild	-10.29	-20.49	< 0.001*	< 0.001*
	Severe vs. mild	-33.48	-31.67	< 0.001*	< 0.001*

Accuracy of deep bite correction

The accuracy of deep bite correction did not differ significantly between the growing and non-growing patients. The median accuracy was 69.0% (IQR, 53.6-77.6) in the growing group and 61.5% (IQR, 55.6-74.1) in the non-growing group (p = 0.633; r = -0.08). In contrast, the severity of deep bite significantly influenced treatment accuracy (p = 0.026; η² = 0.11). The median accuracy was 100.0% (IQR, 54.0-100.0) for mild cases, 66.7% (IQR: 58.6-69.0) for moderate cases, and 57.5% (IQR: 41.7-77.6) for severe cases.

The presence of anterior attachments was not significantly associated with deep bite-correction accuracy (p = 0.288) or posterior attachments (p = 0.969). Maxillary incisor proclination did not demonstrate a statistically significant association with accuracy (p = 0.081). In contrast, mandibular incisor proclination was significantly associated with accuracy of deep bite correction (p = 0.0019; r = 0.52). The median accuracy was 58.6% (IQR, 43.5-74.1) in patients with mandibular incisor proclination compared with 72.0% (IQR, 58.8-100.0) in those without mandibular incisor proclination. Posterior extrusion was not significantly associated with treatment accuracy (p = 0.241). The complete primary analysis of the deep bite correction is presented in Table 4.

**Table 4:** Primary analysis of deep-bite correction accuracy according to patient and treatment characteristics. Data are presented as number (percentage) and median (interquartile range (IQR)). The Mann-Whitney U test was used for comparisons between two independent groups, and the Kruskal-Wallis test was used for comparisons among more than two independent groups. Significant Kruskal-Wallis results were followed by Dunn’s post-hoc test with Bonferroni adjustment. *p < 0.05 was considered statistically significant. η²: eta-squared effect size, r: rank-biserial effect size.

Factor	Category	n (%)	Median (IQR), %	Test statistic	p-value	Effect size
Growth pattern	Growing	25 (50%)	69.0 (53.6-77.6)	U = 337.5	0.633	r = -0.08
	Non-growing	25 (50%)	61.5 (55.6-74.1)			
Overbite severity	Mild	15 (30%)	100.0 (54.0-100.0)	H = 7.305	0.026*	η² = 0.11
	Moderate	19 (38%)	66.7 (58.6-69.0)			
	Severe	16 (32%)	57.5 (41.7-77.6)			
Anterior attachments	Present	20 (40%)	58.8 (53.2-77.6)	U = 246.0	0.288	r = 0.18
	Absent	30 (60%)	70.5 (56.1-98.8)			
Posterior attachments	Present	27 (54%)	69.0 (58.6-77.6)	U = 308.0	0.969	r = 0.01
	Absent	23 (46%)	62.5 (54.6-100.0)			
Maxillary incisor proclination	Present	29 (58%)	61.5 (45.5-77.6)	U = 215.5	0.081	r = 0.29
	Absent	21 (42%)	72.0 (58.6-100.0)			
Mandibular incisor proclination	Present	29 (58%)	58.6 (43.5-74.1)	U = 146.5	0.0019*	r = 0.52
	Absent	21 (42%)	72.0 (58.8-100.0)			
Posterior extrusion	Present	17 (34%)	58.8 (53.6-77.6)	U = 341.5	0.241	r = -0.22
	Absent	33 (66%)	74.1 (69.0-77.6)			

Bonferroni-adjusted post-hoc analysis demonstrated a statistically significant difference in deep bite correction accuracy between mild and severe cases (adjusted p = 0.020). Comparisons between mild and moderate cases (adjusted p = 0.161) and between moderate and severe cases (adjusted p = 1.000) were not statistically significant. The post-hoc analysis is presented in Table 5.

**Table 5:** Bonferroni-adjusted post-hoc pairwise comparisons of deep-bite correction accuracy according to overbite severity. Pairwise comparisons were performed using Dunn’s post-hoc test following a significant Kruskal-Wallis test, with Bonferroni adjustment for multiple comparisons. *p < 0.05 was considered statistically significant. Adjusted p-values are Bonferroni-corrected.

Pairwise comparison	Mean difference	Test statistic	p-value	Adjusted p-value
Mild vs. moderate	16.85	-10.31	0.054	0.161
Mild vs. severe	21.99	13.48	0.007*	0.020*
Moderate vs. severe	5.14	3.17	0.607	1.000

Overall findings

Overall, the severity of the prescribed tooth movement was significantly associated with treatment accuracy for both derotation and deep bite correction. For derotation, significant associations were observed between rotational severity and tooth type, whereas growth status and dental arch were not. For deep bite correction, the severity of the initial deep bite and mandibular incisor proclination was significantly associated with treatment accuracy, whereas growth status, maxillary incisor proclination, and posterior extrusion were not statistically significant.

## Discussion

This retrospective study evaluated the effectiveness of contemporary Invisalign® clear aligners in achieving planned derotation and deep bite correction by comparing the predicted tooth positions with the achieved treatment outcomes. The principal finding was that the severity of prescribed tooth movement was an important determinant of treatment accuracy. Clear aligners have demonstrated effectiveness in managing several types of malocclusions, with outcomes comparable to those of fixed appliances in selected non-extraction cases. However, the available evidence remains limited for complex orthodontic movements, emphasizing the need for further evaluation of the predictability of specific tooth movements using contemporary aligner systems [10].

The absence of a significant difference in derotation accuracy between growing and non-growing patients suggests that contemporary clear aligners can produce comparable rotational corrections across different stages of dental development. This interpretation is supported by a recent age-stratified study that found broadly comparable accuracies of rotational and vertical tooth movements between adolescent and adult patients, although some tooth-specific differences were observed [11]. Although age-related differences in the biological response to orthodontic forces may theoretically influence tooth movement, the relationship between age and tooth movement is complex and may vary according to sex and other patient- and treatment-related factors [12]. More recent evidence also indicates that age, sex, treatment design, staging, attachment characteristics, and the magnitude and direction of prescribed movement can contribute to variability in clear aligner movement predictability [13].

A clear relationship was observed between the severity of rotation and treatment accuracy, with predictability decreasing as the magnitude of required rotational correction increased. This finding is consistent with those of previous studies. Kravitz et al. [14] reported a significant reduction in rotational accuracy for maxillary canines when rotations exceeded 15°, whereas Simon et al. [15] demonstrated that premolar derotation was relatively less predictable, and that the total amount of planned movement significantly influenced treatment efficacy. The reduced predictability with increasing rotational requirements may be explained by the greater biomechanical demands associated with producing and maintaining adequate rotational moment. As the magnitude of programmed movement increases, small discrepancies in aligner fit, tooth-aligner engagement, staging, and force expression may become progressively more consequential. Although contemporary aligner materials and optimized attachments have improved the control of rotational movements, these biomechanical limitations may continue to affect the expression of larger prescribed rotations.

The tooth type was another important factor affecting derotation accuracy in the present study. Differences in rotational predictability between incisors, canines, and premolars have also been reported in previous studies [16]. Kravitz et al. [14] identified canine rotation as a relatively difficult movement to achieve, whereas Simon et al. [15] reported limited predictability for premolar derotation. These findings are further supported by those of Koletsi et al. [16], whose systematic review and meta-analysis demonstrated substantial variation in rotational accuracy according to the tooth type, with maxillary canines showing particularly low predictability. More recently, Stephens et al. [17] reported that prescribed mandibular canine rotation remained under-expressed with Invisalign SmartTrack aligners across different attachment and wear protocols, further highlighting the biomechanical challenges of canine derotation. Such differences may be related to variations in tooth morphology, crown geometry, and the ability of the aligner to generate an effective rotational moment. Although contemporary aligner materials and optimized attachments have improved the control of rotational movements, these biomechanical limitations may continue to affect the expression of programmed rotations.

The present study demonstrated that the severity of the initial deep bite influenced treatment predictability, with mild deep bite corrections showing greater predictability than more extensive corrections. This observation is consistent with recent systematic reviews demonstrating that vertical tooth movements, particularly incisor intrusion, remain among the most challenging movements to achieve accurately with clear aligners [5,6]. Contemporary evidence indicates that deep bite correction is achieved through a combination of true incisor intrusion, incisor proclination, and limited posterior extrusion and that the relative contribution of each mechanism varies according to the treatment plan and malocclusion severity [5]. Therefore, the reduced predictability observed in severe deep bite cases may reflect the increased biomechanical complexity associated with coordinating multiple programmed tooth movements simultaneously. As the magnitude of deep bite correction increases, discrepancies between digitally programmed movement and the clinically achieved outcome become more likely because of limitations in force expression, aligner tracking, and biological response. Similar conclusions have been reported in recent systematic reviews evaluating the effectiveness of clear aligners for vertical correction and complex malocclusions, which emphasized that treatment predictability decreases as movement complexity increases and that evidence for severe deep bite correction remains limited [10,18].

These findings also suggest that mandibular incisor proclination may be an important component of deep bite correction using clear aligners. Proclination of the incisors can reduce the measured overbite by altering the incisor inclination in addition to any true vertical tooth movement. Previous studies have described mandibular incisor proclination and maxillary incisor intrusion among the principal dentoalveolar mechanisms contributing to bite opening with clear aligner therapy, with posterior vertical changes contributing to a lesser extent [6,19]. Therefore, the significant association between mandibular incisor proclination and treatment accuracy observed in the present study may reflect differences in the biomechanical pathway through which bite opening is achieved [20]. When correction depends substantially on changes in incisor inclination rather than true intrusion, the final overbite reduction may be influenced by the magnitude and predictability of programmed angular tooth movement. Contemporary evidence indicates that clear aligners can produce clinically meaningful deep bite correction, but the achieved correction frequently differs from the programmed outcome, particularly when multiple dentoalveolar mechanisms are required [4,5].

The lack of a significant association between posterior extrusion and deep bite-correction accuracy may be clinically meaningful. Posterior extrusion requires careful vertical control, because excessive extrusion may increase lower facial height and induce clockwise mandibular rotation, particularly in patients with undesirable vertical skeletal changes. Contemporary studies on deep bite correction with clear aligners have demonstrated that posterior extrusion can contribute to bite opening, but the magnitude and clinical significance of the associated skeletal changes vary among patients [21,22]. The incorporation of optimized deep bite attachments and other contemporary vertical control features may improve the ability of clear aligners to express programmed posterior movement.

Clinical implications

From a clinical perspective, these findings indicate that contemporary Invisalign® clear aligners can provide useful and reasonably predictable correction of both tooth rotation and deep bite, particularly when required movement is limited. However, clinicians should anticipate reduced predictability when treating severe rotational discrepancies or deeper overbites. Careful case selection, appropriate attachment design, accurate staging, adequate aligner wear, and close monitoring are important. In cases requiring extensive movement, planned overcorrection and timely refinement may be necessary. For deep bite correction, treatment planning should consider whether the desired correction is primarily dependent on true intrusion, incisor proclination, posterior extrusion, or a combination of these.

Limitations

This retrospective design is an important limitation because treatment planning, patient selection, and treatment execution were not prospectively controlled. The study was also based on records obtained from a limited clinical setting and therefore may not be fully generalizable to other populations or treatment protocols. Patient compliance could not be controlled to the same extent as that in a prospective clinical study. Furthermore, the accuracy of digital model superimposition and the quality of available treatment records may influence the measurement of tooth movement. Formal examiner reliability testing, method-error quantification, and blinded outcome assessment were not performed as part of the original retrospective protocol; therefore, measurement and observer-related error cannot be completely excluded.

The relatively small subgroup sizes, particularly for severe movement categories and attachment subgroups, may have limited statistical power and increased the risk of type II error; consequently, non-significant findings should not be interpreted as evidence of clinical equivalence or lack of influence. Attachment configuration was determined according to the treating clinician’s judgment rather than a standardized protocol, introducing potential heterogeneity and confounding. The accuracy of digital model superimposition may also be influenced by the stability of the selected reference structures, although minimal molar movement in all three spatial planes was used as a predefined eligibility criterion. This study focused on the original aligner sequence and therefore did not assess the additional corrections that may subsequently be obtained through refinement treatment. Finally, differences in individual biological responses and clinician treatment planning may have contributed to variation in treatment outcomes. These limitations should be considered when interpreting the findings and applying them in routine clinical practice.

## Conclusions

Contemporary Invisalign® clear aligners demonstrated good overall effectiveness for derotation and deep-bite correction. Treatment accuracy was influenced primarily by the severity of the required movement, with greater discrepancies observed as treatment demands increased. Tooth type also affected derotation predictability, whereas mandibular incisor proclination influenced deep-bite correction. No statistically significant differences were observed according to growth status or dental arch; however, these findings should be interpreted cautiously given the subgroup sample size and variability in measurements. These findings support careful case selection, appropriate biomechanical planning, and consideration of overcorrection or refinement for complex movements.
